# M2 Muscarinic acetylcholine receptor modulates rat airway smooth muscle cell proliferation

**DOI:** 10.1186/1939-4551-6-22

**Published:** 2013-12-30

**Authors:** Fabiola A Placeres-Uray, Christopher A Febres-Aldana, Ruth Fernandez-Ruiz, Ramona Gonzalez de Alfonzo, Itala A Lippo de Becemberg, Marcelo J Alfonzo

**Affiliations:** 1 Sección de Biomembranas, Instituto de Medicina Experimental, Facultad de Medicina, Universidad Central de Venezuela (U.C.V), Caracas, Venezuela

**Keywords:** Airway smooth muscle, Muscarinic receptors, Carbamylcholine, ASMC inhibition

## Abstract

Airways chronic inflammatory conditions in asthma and COPD are characterized by tissue remodeling, being smooth muscle hyperplasia, the most important feature. Non-neuronal and neuronal Acetylcholine acting on muscarinic receptors (MAChRs) has been postulated as determinant of tissue remodeling in asthma and COPD by promoting proliferation and phenotypic changes of airway smooth muscle cells (ASMC). The objective was to evaluate proliferative responses to muscarinic agonist as carbamylcholine (Cch) and to identify the MAchR subtype involved. ASMC were isolated from tracheal fragments of Sprague–Dawley rats by enzymatic digestion. Proliferation assays were performed by MTS-PMS method. Viability was confirmed by trypan blue exclusion method. Mitogens as, epidermal growth factor (EGF), Tumor necrosis factor-alpha (TNF-α) and fetal bovine serum (FBS) increased ASMC proliferation (*p* < 0.05, n = 5). Cch alone increased ASMC proliferation at 24 and 48 hrs. However, combination of Cch with other mitogens exhibited a dual effect, synergistic proliferation effect in the presence of EGF (5 ng/mL) and 5% FBS and inhibiting the proliferation induced by 10% FBS, EGF (10 ng/mL) and TNF-α (10 ng/mL). To determine the MAChR subtype involved in these biological responses, a titration curve of selective muscarinic antagonists were performed. The Cch stimulatory and inhibitory effects on ASCM proliferation was blocked by AF-DX-116 (M_2_AChR selective antagonist), in greater proportion than 4-DAMP (M_3_AChR selective antagonist), suggesting that the modulation of muscarinic agonist-induced proliferation is M_2_AChR mediated responses. Thus, M_2_AChR can activate multiple signal transduction systems and mediate both effects on ASMC proliferation depending on the plethora and variable airway microenvironments existing in asthma and COPD.

## Background

Chronic inflammatory conditions of the airways are usually associated with the development of structural changes of the airways; a phenomenon commonly described as airway remodeling. This process is seen in both asthma and Chronic Obstructive Pulmonary Disease (COPD), albeit the nature, localization and extent of the remodeling are variable [1-4]. Airway remodeling is progressive and the degree of structural changes correlates with disease severity [4]. In this sense, the Airway Smooth Muscle Cells (ASMC) hyperplasia has been postulated as the main mechanism of airway smooth muscle thickening [5,6].

ASMC are multifunctional cells that have high phenotypic plasticity. These cells can shift between different phenotypes depending on the stimulation conditions. Accordingly, mitogenic factors can induce the reversible transition to “synthetic-proliferative” phenotype, characterized by high capacity for cell proliferation and secretion [7]. Several mediators, such as growth factors, cytokines, extracellular matrix components, and G protein-coupled receptors (GPCR) agonists have been found in bronchoalveolar lavage fluid of asthmatics. Among the mediators identified include epidermal growth factor (EGF) and tumor necrosis factor-alpha (TNF-α) [8] and acetylcholine [9].

EGF binds to receptors with intrinsic tyrosine kinase activity [10]. All EGF receptor (EGFR) subtypes are express by ASMC. However, that exerts its effect by acting on the family 1 and 2 [10,11]. Furthermore, this growth factor has been shown as the most potent for ASMC proliferation stimulation. EGFR activation in ASMC triggers mitogenic pathways through p21Ras and PI3-K, resulting in phosphorylation at serine and threonine residues of several transcription factors by mitogen-activated protein kinases (MAP kinases). Thus, DNA synthesis is promoted and initiates the cell cycle [10,12].

The biological effects of TNF-α are mediated through two receptors of similar affinity (TNF-R1, CD120a; 55-kd and TNF-R2, CD120b, 75-kD) [13]. In experimental models have been demonstrated their contribution to chronic inflammation and airway hyperresponsiveness mediated by TNF-R1 [14-16]. ASMC express both TNF-R subtypes [15], whose stimulation induces an increase of proliferation either directly or through other mediators [17]. In this sense, TNF-α can induce MAPK pathway activation including ERKs, p38 MAPK and JNK [12,15,16].

Acetylcholine (Ach) is agonist of muscarinic receptors (MAchRs) that traditionally associated with airway smooth muscle contraction and mucus secretion. Recently it has been postulated as determinant of bronchial remodeling [18]. There are two sources of Ach in the airways: 1) neural, provided by parasympathetic fibers, from vagal nerve and 2) non-neural, from airway epithelium and inflammatory cells present in the chronic inflammation process, currently found in asthma [19]. ASMC express two sub-types of MAChRs: Muscarinic receptor type 2 (M_2_AChR) and muscarinic receptor type 3 (M_3_AChR), whose activation promote synthetic functions, proliferation and phenotypic changes depending on stimulation conditions [19-21]. ASMC proliferation induced by ACh is reversed by the muscarinic antagonists, such as tiotropium bromide [22]. ACh and others agonists of GPCR are not able by themselves to stimulate ASMC proliferation, but enhance the action of growth factors by different signal pathways [23]. However, the results differ between different animal models. Thus, the aim was to study in rat ASMC, the proliferative responses to a muscarinic agonist such as carbamylcholine (Cch), and to establish the MAChR subtype involved. In addition, to evaluate as Control, the classic mytogenic responses induced by fetal bovine serum (FBS), EGF and TNF-α. A preliminary description of this work has been reported [24].

## Methods

ASMC were obtained from tracheas of female Sprague–Dawley rats (12–14 weeks, weighing between 300–350 g) from animal facility of the Instituto de Medicina Experimental (I.M.E) of the Universidad Central de Venezuela (U.C.V). The animals were maintained according to international standards for animal care and experimental protocol was approved by the Bioethics Committee of I.M.E.

### Isolation and culture of rat airway smooth muscle cells

Primary cultures of rat ASMC were established as previously reported [25-27]. Rat trachea was dissected in ice-cold phosphate- buffer saline (PBS) solution, pH 7.4 (composition in g/dL: 0.2 KH_2_PO_4_, 0.8 NaCl, 1.15 Na_2_HPO_4_). The epithelium was removed, and muscle were gently separated from underlying connective tissue in small bundles, which were placed in digestion solution (Ringer plus Ca^2+^) containing 4 mg/mL collagenase II (Worthington®, UK) and 0.6U/mL dispase (Gibco®, USA) by 50–60 min at 37°C under 5% CO_2_ Atmosphere. The cell suspension was centrifuged at 500xg by 15 min. Cell suspensions and explants were separately incubated in 25-cm^2^ flasks at 37°C in a humidified atmosphere of 5% CO2 for 16–24 days (incubator NUAIRE™). The cells were cultured in Dulbecco’s modified Eagle’s medium/F-12 (DMEM/F-12; Gibco®, USA) supplemented with 10% FBS (Gibco®, USA), 1% L-Glutamine (Gibco®, USA), 2% penicillin/streptomycin (Gibco®, USA), 2% amphotericin (Gibco®, USA) and were passaged when confluent using trypsin/EDTA (0.5 g/L porcine trypsin, 0.2 g/L EDTA, 4Na/L) of Hanks’ balanced salt solution (HBSS); Sigma®, USA. Cells between passages 3 and 7 were used for all experiments.

### Proliferation assays

ASMC proliferation was estimated by the nonradioactive method (MTS-PMS assay) based on the formation of tetrazolium salts [28] using, CellTiter 96® AQueous (Promega®, USA). A fixed number of cells were seeded onto 96 wells plates. After 24 hrs, culture medium was changed by medium without FBS to equilibrate cell cycle in G_0_/G_1_ phase. After 12–24 hrs, cells were incubated at 37°C and 5% CO_2_ with solution containing the treatment according each experimental conditions using the following compounds: AF-DX-116 (Tocris®, USA), 4-DAMP (Tocris®, USA), carbamylcholine chloride (Cch; Sigma®, USA), human recombinant EGF (Chemicon International®, USA), rat recombinant TNF-α (Chemicon International®, USA)]. After, the exposure time finished, 100 μL of medium plus 10 μL MTS [3-(4,5-dimethylthiazol-2yl)-5-(3-carboxymethoxyphenyl)-2-(4-sulfophenyl)-2H-tetrazolium]-PMS [Phenazine Methosulfate]/well, and incubated for additional 90 min. The cell proliferation were determinated measuring optical density (OD) at λ = 492 nm, which is proportional measure to the number of viable cells in wells.

### Trypan blue dye exclusion assay

Proliferation and viability was confirmed by trypan blue 0.1% exclusion method [29]. A fixed number of cells were seeded onto 6 wells plates with 2 mL of medium for 24 hrs. Then, culture medium was changed by non-supplemented medium to equilibrate cell cycle in G_0_/G_1_ phase. After 12–24 hrs, cells were incubated at 37°C and 5% CO_2_ with solution containing each compound as experimental condition described. After 72 hrs, the cells were detached using trypsin/EDTA and centrifuged at 500xg for 15 min. The cells were vigorously resuspended in 1 mL of medium. The number of cells was estimated in a mixture of 10 μL of cell suspension plus 90 μL of 0.4% trypan blue [dilution factor (DF) 1:10] by 5 min, then 20 μL of this mixture was placed in the hemocytometer and observed under light microscopy. All cells in central quadrant and four quadrants of corners were counted. Cells number was estimated and the viability was determined (considering that only dead cells capture the dye) using the equations previously described [30].

### Statistical analysis

Values reported for all data are means ± SE. The statistical significance of differences between means was determined by an unpaired two-tailed Student’s *t*-test. Differences were considered to be significant as *p* < 0.05. The parameters log IC_50_ ± SE were estimated using the GRAPH PAD® program.

## Results and discussion

### Rat ASMC proliferation in response to mitogens

**Figure 1:**
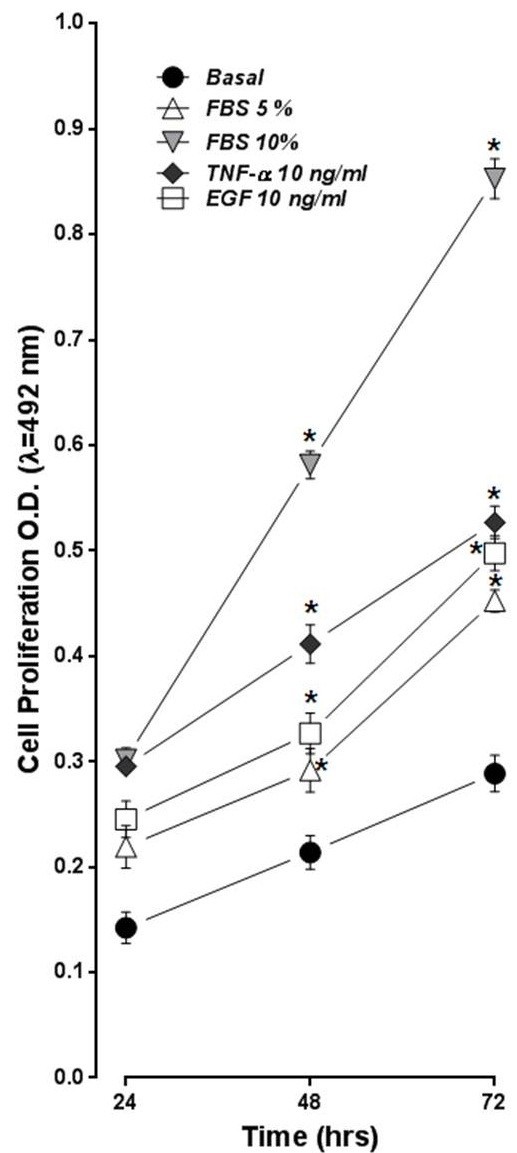
**Rat ASMC proliferation in response to FBS, EGF and TNF-α.** ASMC (1 × 10^3^ cells/well) were cultured in 96 wells plates at 37°C/5%CO_2_ for 24, 48 and 72 hrs in a medium without FBS (basal; ●), in presence of 5% FBS (∆), 10% FBS (▼), EGF 10 ng/mL (□), TNF-α 10 ng/mL (♦). Cell proliferation was determined using a colorimetric method (MTS-PMS), measuring Optical Density (O.D.) at λ = 492 nm. Data is the mean ± ES of n = 5 experiments for triplicate. The proliferation responses of each mitogen concentration vs. basal was significant (*) *p* < 0.05.

Rat ASMC were incubated by 24, 48 and 72 hrs at 1 × 10^3^cells/well in 96 well plates to evaluate its basal proliferation and response to mitogens using MTS-PMS method as shown in Figure 1. Basal proliferation of ASMC was increased time-dependent manner being significant at 24, 48 and 72 hrs. Mitogens increased ASMC proliferation at all times of culture tested. The mitogenic effectiveness at 72 hrs was the following: 10%FBS > 5%FBS = EGF (10 ng/mL) = TNF-α 10 ng/mL (n = 5, *p* < 0.05). In addition, EGF and TNF-α, showed a dual effect increasing ASMC proliferation reaching maximum effect at 10 ng/mL and higher concentration the proliferation effect decreased (data not shown).

**Figure 2:**
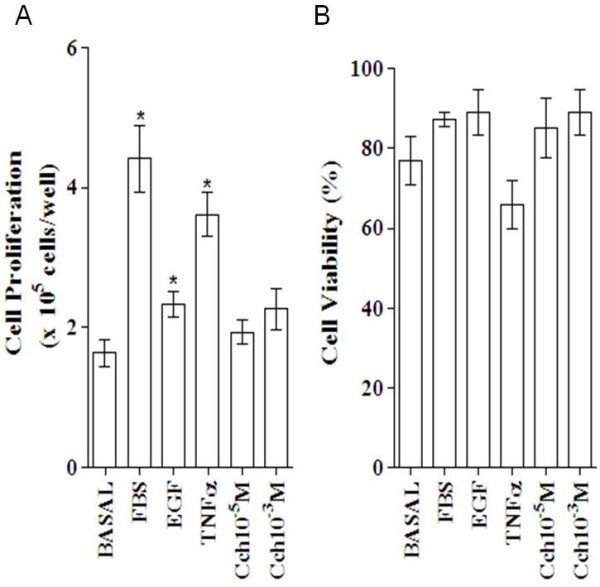
**Rat ASMC proliferation (A) and viability (B) in presence of mitogens and Cch.** ASMC (2 × 10^5^ cells/well) were cultured in 6 wells plates at 37°C/5%CO_2_ for 48 hrs in presence of DMEM/F-12 medium (basal), 10% FBS, EGF 10 ng/mL, TNF-α 10 ng/mL and Cch 10^-5,-3^ M. Cell proliferation and viability were determined using Trypan blue dye exclusion method. Data is the mean ± ES of n = 5 experiments for triplicate. The proliferation responses of each mitogen concentration vs. basal was significant (*) *p* < 0.05.

Viability and cell proliferation were also evaluated by trypan blue dye exclusion method as shown in Figure 2. In these assays, ASMC were culture in 6 well plates at 2x10^5^cells/well and mitogens as FBS, EGF, TNF-α, which stimulated ASMC proliferation. However, there was not a clear cut correlation between cell viability with cell proliferation. In this sense, TNF-α (10 ng/mL) increased cell proliferation but decreased cell viability (n = 5, p < 0.05), indicating that TNF-α induces cell division and also promotes cell death (apoptosis). The mechanism of cell death induced by TNF-α could not be assessed with the methodology here applied.

ASMC proliferation has been studied for in several conditions using various experimental animal and human models. Fetal Bovine Serum (FBS) is a potent mitogenic, that effect has been explained by the Reactive oxygen species (ROS) generation [31]. Thus, exposing normal ASMC to FBS induced proliferation, which can trigger signal transduction leading to gene expression [32,33]. H_2_O_2_ treatment of airways myocytes successively stimulates the MAP kinase superfamily members, which are important in transduction of mitogenic signals to the nucleus [34]. This has important implications for the pathogenesis of remodeling in the asthmatic airways, where myocytes are exposed to ROS from activated eosinophils/neutrophils and macrophages that are present during the acute and chronic inflammation process presents in asthma and COPD. In addition, even serum can leaks from vascular capillary system as a consequence of submucosal edema and increased submucosal vascular permeability in asthma [35]. In our experiments, FBS was the best mitogen for rat ASMC, which confirmed the biological actions previously reported.

Another mitogen studied was Tumor necrosis factor (TNF-α), which is a potent proinflammatory cytokine and its role as a potential mediator in asthma has been well described [36,37]. Moreover, TNF-α has been reported to be a poor mitogen and it can also modulate cultured ASM cells to proliferate [38,39]. In our study, these reported biologic actions of TNF-α on rat ASMC were confirmed.

EGF is a mitogen that been reported to stimulate ASMC growth *in vitro*[40] and this growth factor has been shown to be upregulated in asthmatic human airways [41]. In our work, we found that EGF was able to stimulate rat ASMC proliferation and confirmed the reported findings. In summary, both TNF-α and EGF displayed similar mitogenic activity in rat ASMC. In addition, both mitogens exhibited a dual effect on ASMC proliferation.

## Muscarinic agonist (Cch) modulates rat ASMC proliferation

### The effects of muscarinic antagonists AF-DX-116 and 4-DAMP on this Cch modulation

**Figure 3:**
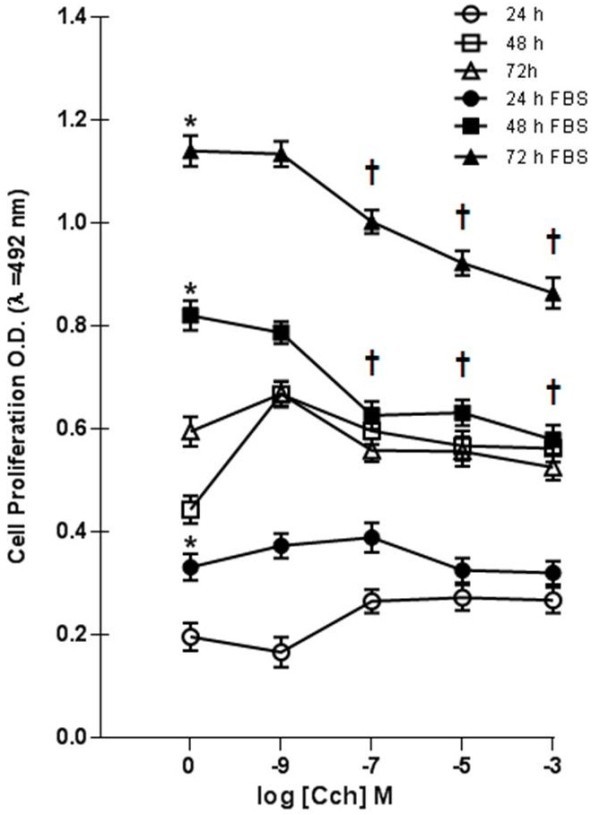
**Rat ASMC proliferation response to muscarinic agonist Cch.** ASMC (1 × 10^3^ cells/well) were cultured in 96 wells plates at 37°C/5% CO_2_ with increasing concentration of muscarinic agonist Cch in medium without FBS during 24 (○), 48 (□), y 72 (∆), and 10% FBS, during 24 (●), 48 (■), y 72 (▲) hrs. Cell proliferation was determined using a colorimetric method (MTS-PMS), measuring O.D at λ = 492 nm. Data are the mean ± SE of n = 6 experiments for triplicate. Mitogen responses of 10% FBS vs. basal was significant (*) *p* < 0.05. The inhibitory effect of Cch was significant (┼) *p* < 0.05.

Rat ASMC were incubated by 24, 48 and 72 hrs at 1x10^3^cells/well in 96 well plates with increasing doses of Cch in the presence and absence of 10% FBS (Figure 3). Basal proliferation increased, in a dose dependent manner by Cch, being significant at 24 and 48 hrs (n = 6, *p* < 0.05) (Figure 3). However, Cch, decreases the ASMC proliferation induced by 10% FBS (Figure 3). Thus, a dose-dependent inhibitory effect on ASMC proliferation by Cch was significant at 48 and 72 hrs. Proliferation inhibition was not due to death cell because ASMC viability in presence of Cch was confirmed with blue dye exclusion method (Figure 2).

**Figure 4:**
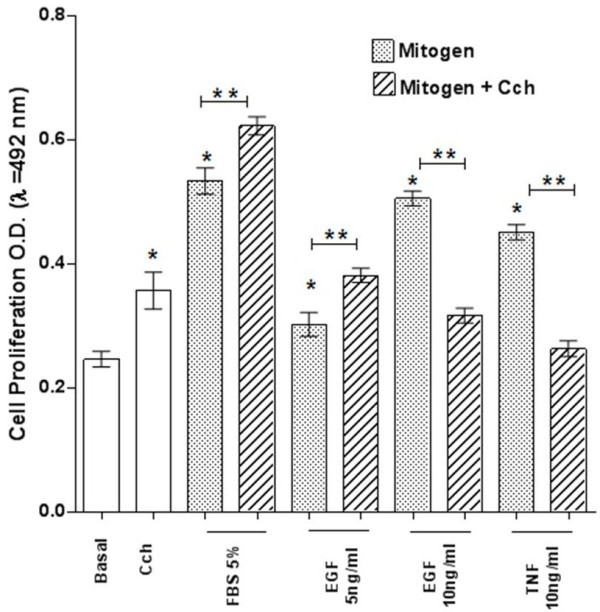
**Rat ASMC proliferation in response to Cch and mitogens.** ASMC (1 × 10^3^ cells/well) were cultured in 96 wells plates at 37°C/5% CO_2_ with DMEM/F-12 medium (basal), Cch 10^-3^ M, 5%FBS, EGF 5 and 10 ng/mL, and TNF-α 10 ng/mL during 24 hrs. Cell proliferation was determined using a colorimetric method (MTS-PMS), measuring O.D at λ = 492 nm. Data is the mean ± ES of n = 6 experiments for triplicate. Each mitogen proliferation responses vs basal was significant (*) *p* < 0.05. Moreover, significant differences (**) *p* < 0.05 between mitogen vs mitogen plus Cch condition were found.

To further evaluate the modulation properties of Cch on ASCM proliferation, it was found that, at 24 hrs, Cch stimulated cell proliferation and synergistically potentiated the mitogenic effect of 5% FBS and a similar trend was observed with EGF 5 ng/mL (n = 6, *p* < 0.05) (Figure 4). Nonetheless, the Cch inhibitory effect was observed with EGF 10 ng/mL and TNF-α 10 ng/ml being significant (n = 6, *p* < 0.05) (Figure 4).

**Figure 5:**
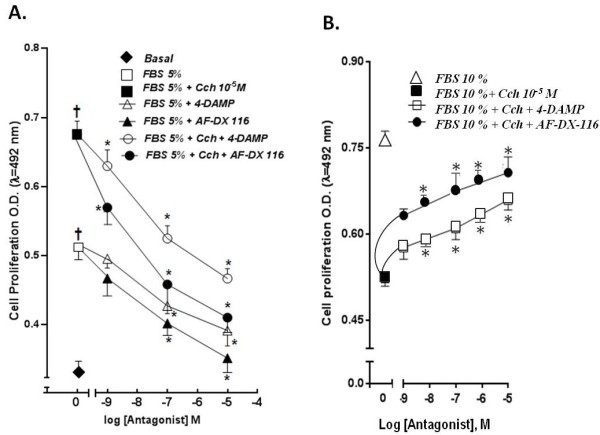
**Effect of selective muscarinic antagonists AF-DX-116 (M**_**2**_**AChR) and 4-DAMP (M**_**3**_**AChR) on ASMC proliferation at 5 and 10% FBS. A**. Effect of increasing concentration of AF-DX-116 and 4-DAMP on the synergistic effect induced by Cch in presence of 5% FBS. ASMC (1× 10^3^ cells/well) were cultured in 96 wells plates for 72 hrs. Cell proliferation was determined using a colorimetric method (MTS-PMS), measuring O.D at λ = 492 nm. (n = 5, for triplicate). The synergistic proliferation responses in the presence of 5% FBS and Cch (1 × 10^-3^ M) plus 5% FBS against basal condition were significant different (┼) *p* < 0.05. The muscarinic antagonists inhibitory responses were significant against the “0” Cch condition (*) *p* <0.05. The estimated Log IC_50_ ± SE were 4-DAMP + FBS = −7.34 ± 0.21; 4-DAMP + FBS + Cch = −7.38 ± 0.42; AFDX-116 + FBS = −7.26 ± 0.51 and AFDX-116 + FBS + Cch = −8.99 ± 0.45. **B**.- Effect of increasing concentrations of AF-DX-116 and 4-DAMP on the anti-proliferative effect induced by Cch (1x10^-5^ M), in presence of 10% FBS. ASMC (1 × 10^3^ cells/well) were cultured in 96 wells plates for 72 hrs. Cell proliferation was determined using a colorimetric method (MTS-PMS), measuring O.D at λ = 492 nm. (n = 5, for triplicate). The muscarinic antagonists stimulatory proliferative responses were significant (*) *p* < 0.05 against the anti-proliferative Cch (1x10^-5^ M). The estimated Log IC_50_ ± SE were 4-DAMP = −7.11 ± 0.71 and AFDX- = −9.40 ± 0.37.

To determine the MAchR subtype involved in these opposite effects displayed by Cch. ASMC at 1x10^3^cells/well were incubated in 96 well plates in the presence of 5% FBS and 5% FBS plus Cch leading to a rise in cell proliferation as exhibited in Figure 5. Under these experimental conditions, ASMC were exposed to increasing concentrations of preferential muscarinic antagonist 4-DAMP (for M_3_AChR) and AF-DX-116-DS (for M_2_AChR) as shown in Figure 5A. These muscarinic antagonists reversed both, the 5% FBS induced proliferation activity and the Cch-induced proliferation (n = 4, *p* < 0.05) as shown in Figure 5A. Unexpectedly, the 5% FBS induced proliferation was inhibited by these muscarinic antagonists displaying, the log IC_50_ for 4-DAMP and AFDX-116, which were similar as 4-DAMP + FBS = −7.34 ± 0.21 AFDX-116 + FBS = −7.26 ± 0.51. These results may be explained by some inverse agonist actions described by these muscarinic drugs [42]. This experimental findings may be a research subject in the future.

Interestingly, in the case of the synergistic Cch and FBS proliferation effect, both muscarinic antagonists were able to inhibit such proliferation activity displaying different values of the log IC_50_ for the 4-DAMP + FBS + Cch = −7.38 ± 0.42 and AFDX-116 + FBS + Cch = −8.99 ± 0.45. From these data, there is two order of magnitude of difference between these values supporting a pharmacological profile that AFDX 116 > 4-DAMP, that belongs to an M_2_AchR.

Trying to understand this novel finding on the ability of Cch to exhibit anti-proliferative properties especially at high mitogen concentration (10% FBS). It was found, this anti-mitogenic Cch effect was reversed, in a dose-dependent manner, by preferential muscarinic antagonists as AF-DX-116 (M_2_AChR antagonist), which was more efficient than 4-DAMP (M_3_AChR antagonist) to reverse this novel Cch inhibition activity as shown in Figure 5B. The proliferative stimulatory responses displayed by muscarinic antagonists reversed significantly the anti-mitogenic Cch (1x10^-5^ M) action (*p* < 0.05). Thus, the Log IC_50_ ± SE were 4-DAMP = −7.11 ± 0.71 and AFDX- = −9.40 ± 0.37 were estimated. The difference between these log IC_50_ values is more than 2 orders of magnitude, that support a pharmacological profile is AFDX 116 > > 4-DAMP clearly belongs to an M_2_AchR.

It important to point out that all Cch effects here described on rat ASMC proliferation were affected by muscarinic antagonists, which supported the rationale that these mitogenic and anti-mitogenic effects are mediated, via muscarinic receptors, and nor through nicotinic receptors, which may be affected by Cch.

Interestingly, in our study model rat ASMC, muscarinic agonist, Cch, displayed three distinct effects on ASMC proliferation: 1) stimulatory effect by itself, 2) synergistic effect, at low concentrations of mitogens (5% FBS, EGF 5 ng/mL), and 3) inhibitory effect, at maximum concentration of mitogens (10% FBS, 10 ng/mL of EGF or TNF-α) as above described.

It is complex matter to explain these diverse biological effects that are initiate by the binding of a neurotransmitter (ACh) to muscarinic receptors (GPCR) at sarcolema of ASMC, with the involvement of some intracellular seconds messengers (cGMP, cAMP, Ca^2+^) that trigger several intracellular signal cascades that cross-talk involving protein-protein interactions and reversible posttranslational modifications (phospho/dephosphorylation processes) to activate or regulate the nuclear factors involve in the DNA duplication and cell division, which is a fast growing research field in the last 20 years.

Neuronal and non-neuronal ACh, has been proposed to promote airway remodeling and increased smooth muscle thickening and airway hyperresponsiveness development in asthma models, which was prevented by a specific antagonist of M_3_AChR such as tiotropium bromide [19,43]. Classically, it has been claimed that agonist muscarinic stimulation “in vitro” is not sufficient to induce ASMC proliferation and only in combination with growth factors, the mitogenic effect was observed [19,21,23]. In this sense, we found a stimulatory effect on rat ASMC proliferation by Cch, which was less potent than FBS, EGF and TNF-α. Muscarinic stimulation has been associated with MAPK and PI3-K activation [19-21]. Moreover, ERKs activation and phosphorylation is reaching in response to agonist of M_2_AChR and M_3_AChR. The M_3_AChR pathway is linked to a G_q_ protein mediated and dependent of Raf-1 phosphorylation by PKC, whereas M_2_AChR pathway is G_i/o_ protein mediated and depends on PI3-K activation [43]. However, in human and bovine ASMC, MAChR proliferative activation as well as others GPCRs requires a growth factor to activate MAPK in sustained manner [17,43]. Thus, gene transcription associated with cell cycle promotion is not sufficient to enable the transition G_0_ phase to G_1_ phase.

Muscarinic agonist synergistic effect on rat ASMC proliferation in the presence of low doses of EGF and 5% FBS was observed. The co-administration of muscarinic agonists with EGF in human ASMC induces a synergistic proliferative stimulus. This effect was associated with sustained activation of p70 S6 kinase [21,44], an effect mediated by Gq derived Gβγ subunits that activate phosphatidylinositol-3-kinase (PI3K) in concert with the EGF receptor [19,44]. In line with these findings, muscarinic receptor agonists induce an increase in proliferation of airway smooth muscle cells in combination with platelet-derived growth factor (PDGF), which is mediated by Gq-protein-coupled M_3_AChR and appears to involve a synergistic inhibitory phosphorylation of glycogen synthase kinase-3 (GSK-3) [45].

Similar synergistic effects of muscarinic agonists in human and bovine ASMC are antagonized by 4-DAMP and DAU 5884, in consequence appears to be M_3_AChR mediated [19]. However, our data obtained from rat ASMC incubated with preferential muscarinic antagonists AF-DX-116 (for M_2_AChR) [46] and 4-DAMP (for M_3_AChR) suggest that synergistic effect is mediated through the activation of M_2_AChR (log IC_50_ AF-DX-116 < IC_50_ 4-DAMP). Our results suggest that constitutive activity of M_2_AChR could be necessary to maintain ASMC proliferation in 5% FBS response. Additionally, M_2_AChR is coupled to a pertussis toxin (PTX) sensitive G_i/o_ protein. Consequently, in several studies have been reported that mitogenic effect of Cch on human ASMC is antagonized by PTX [47,48]. To explain these synergists effects, a PTX sensitive signal cascade involving p21Ras and MAPK activation induced by muscarinic agonists may be consider [49] as reported, in vascular smooth muscle cells, where PTX treatment decreased basal proliferation and response to FBS and PDGF [50]. Interestingly, growth factors induce activation and dissociation of heterotrimeric G proteins in many cell types, either through direct (EGF → G_i/o_, G_s_, PDGF → G_i/o_) or indirectly interaction (PDGF/EDG1 → G_i/o_) [51]. Src and PI3-K activation by PDGF in bovine ASMC is mediated by a PTX-sensitive G protein [52]. Therefore, it is possible that M_2_AChR/G_i/o_ protein/p21Ras and MAPK signal cascade in response to Cch might be more efficient to potentiate the signal transduction of growth factors present in FBS. Nonetheless, synergistic effect of muscarinic agonists may involve a sustained activation of p70^S6K^ in promotion of protein synthesis related with cell cycle transition [21,44] rather than increased of MAPK activation [44]. Moreover, glycogen synthase kinase-3β (GSK-3β) could be another possible mediator due to it has been involved in methacholine (muscarinic agonist) synergism on human ASMC proliferation. Thus, an active form of GSK-3β (dephosphorylated) inhibits cell proliferation through negative regulation of some mitogenic promoters such as cyclin D1 accumulation in cell nucleus [45].

Briefly, muscarinic agonist synergism with other mitogens on rat ASMC proliferation could be mediated by interactions between several pathways and signal transduction effectors as GSK-3β, p70^S6K^ and ERKs. During the preparation of this manuscript, it was reported that muscarinic agonists (metacholine) exhibits synergistic effects on TGF-β1-induced proliferation, which were reduced by tiotropium and the M_2_AChR subtype antagonist gallamine, but not the M_3_AChR antagonist DAU5884. Moreover, pertussis toxin treatment also prevented the potentiation of TGF-β1-induced proliferation by methacholine, via an M_2_AChR coupled to Gi/o protein. These authors concluded that exposure to TGF-β1 induces ASMC proliferation, which is enhanced by M_2_AChR stimulation [53]. These explanations on the role of M_2_AChR are similar to the ones here described for rat ASMC.

Muscarinic agonist inhibition of rat ASMC proliferation is an original experimental finding of this work. This effect was observed when ASMC were incubated with Cch plus mitogens at its maximum doses for proliferation responses such as 10% FBS. This inhibitory novel response seems to be also mediated by M_2_AChR from pharmacological profile responses (log IC_50_ AF-DX-116 < log IC_50_ 4-DAMP).

Muscarinic agonist inhibition mechanism may be the result of the activation of two signaling pathways: 1) The cGMP/PKG activation cascade [54-58], and 2) MAPK activation: p38 MAPK and JNK cascade [11]. Increased cGMP production by muscarinic agonist (Cch) has been reported previously in bovine tracheal smooth muscle [54-56]. Transduction mechanisms proposed include MAchR stimulation, G protein activation and subsequent activation of NO-sensitive-soluble guanylyl cyclase (NO-sGC) and/or membrane-spanning Natriuretic Peptide Receptor guanylyl cyclase (NPR-GC). Recently, we have described a M_2_AChR coupled to a G_i/o_ protein-dependent process, that augmented NO-sGC activity in bovine smooth muscle, independently of nitric oxide (NO) [54,56]. Likewise, M_3_AChR/G_q_ protein complexes have been associated with the NPR-GC-B activation [54,56] producing cGMP, which can activate PKG. This last nucleotide-dependent kinase can phosphorylate transcription factors associated with inhibition of gene expression that promote cell cycle, also induce increment of proteins that leads cell cycle arrest as p21^Cip1/Waf1^[58].

The involvement of these two GCs (sGC and/or NPR-GC-B) in rat ASMC in response to Cch is supported by some additional experimental evidences (data not shown): 1) Cch induced proliferation is blocked by sodium nitroprusside (SNP; NO donor). 2), Cch blocked synergistic effect on ASMC proliferation induced by ODQ, a selective inhibitor of NO-sGC, and 3) Cch potentiates the inhibitory effect of natriuretic peptide type-C (CNP; activator of NPR-GC-B) on ASMC proliferation. All these additional data suggest that inhibitory effect of Cch on mitogen-induced ASMC proliferation could include the activation of ones of these cGMP-dependent signaling pathways above mentioned. The identification of the members of the cGMP-PKG cascades related to this M_2_AChR-dependent inhibitory effect on ASMC proliferation is under intense investigation in our laboratory.

The fact that ASMC proliferation inhibition by Cch was observed at high doses of mitogens leads to ask whether inhibition is due to certain level of mitogenic pathways activation, which are associated in parallel with anti-proliferative pathway facilitation (e.g. ↑[cGMP]_int_) or mitogenic pathways over-activation (e.g. p38MAPK or JNK) trigger cell death [11,59]. Inhibition of cell proliferation by muscarinic agonists has been reported in ovarian cells [46], NIH3T3 cells [60], and cancer cells [61], in most cases M_3_AChR and p21^Cip1 Waf1^ expression was involved in cell cycle arrest. The dual effects of Cch on proliferation have also been reported for others cell systems [62].

ASMC phenotype present in these assays may be another factor that can influence these results. ASMC express both M_2_AChR and M_3_AChR, in a proportion that depends of species and cell phenotype. In ASMC with contractile phenotype predominates M_3_AChR expression (~80%) [37]. Prolonged serum deprivation induces M_3_AChR transcription and expression in ASMC that express contractile proteins and generate a basal lamina rich in laminina. Airway remodeling models suggest that M_3_AChR mediated ACh effects “in vivo” [17-20]. Chronic activation of M_3_AChR may constitute an “in vivo” mechanism for ASMC phenotype modulation, which is crucial event in tissue remodeling rather proliferation stimulation [18,28]. By other hand, ASMC with synthetic-proliferative phenotype express mainly M_2_AChR [63], which seem to be our case. Therefore, cell population used in this work was heterogeneous in similar manner to ASMC “in vivo” [7]. Thus, the M_2_/M_3_AChR ratio is 4:1, which has been described in intact airway smooth muscle cells [64,65].

The exact cellular mechanisms underlying MAChR-modulated DNA synthesis and ASMC proliferation are not fully understood. ASMC proliferation involves several intracellular pathways leading to DNA replication and cell division as above discussed. In relation to this novel inhibitory effect of muscarinic agonists here described may improve our understanding of the intracellular mechanisms underlying the activation of mitogenic and anti-mitogenic pathways and provide insights for therapeutic drug development. Recent evidence suggests that ACh acting through muscarinic receptors may play an inhibitory role in the airway remodeling. The anticholinergic drug tiotropium bromide, which selectively antagonizes the M_3_AChR subtype, could be beneficial in attenuating airway remodeling in chronic asthma [66]. These authors reported that in murine (BALB/c mice) models of chronic asthma, sensitized and challenged to ovalbumin, the expression of the M_3_AChR was inhibited and the M_2_AChR was elevated by the administration of tiotropium bromide. Our results on the role of M_2_AChR inhibiting the mitogen-induced proliferation may be relevant, which may be similar environment to the ones present in chronic asthma, which can explain these interesting experimental results of tiotropium bromide on M_3_/M_2_AChR expression [66].

## Conclusions

Muscarinic agonist has three effects on “in vitro” rat ASMC proliferation: 1) stimulation, 2) synergism, and 3) inhibition. Interestingly, both biological actions seem to be mediated by M_2_AChRs through activation of distinctive and multiple signal transduction pathways, which may depend on the cell phenotype and the type and mitogen concentration used. These findings are important in ASMC proliferation induced by ACh “in vivo” especially on cells with synthetic-proliferative phenotype. If, mitogenic and anti-mitogenic effects are both mediated by the same receptor, leads us to propose an ASMC proliferation modulation by ACh, via M_2_AChRs. Our data here reported support the rationale about the need for the developing of new muscarinic antagonists-derivated from tiotropium bromide or similar compound that preferentially antagonize the putative M_3_AChR involves in the mitogen-induced ASMC proliferation associated to the airway remodeling presents in chronic asthma and COPD and increase the level of expression of this novel action of M_2_AChR acting as anti-proliferation receptor.

## Abbreviations

ACh: Acetylcholine; ASMC: Airway smooth muscle cells; Cch: Carbamylcholine; COPD: Chronic obstructive pulmonary disease; cGMP: Cyclic guanosine monophosphate; EGF: Epidermal growth factor; EGFR: EGF Receptor extracellular signal-regulated kinases; ERKs: Fetal bovine serum; FITC: Fluorescein-1-isothiocyanate-conjugated; GPCR: G protein-coupled receptors; GSK-3 β: Glycogen synthase kinase-3β; IC50: half maximal inhibitory concentration; MAPK: Mitogen-activated protein kinases; mAchR: Muscarinic receptors; OD: Optical density; NO: Nitric oxide; NPR-GC: Natriuretic peptide receptor sensitive guanylyl cyclase; PDGF: Platelet-derived growth factor; PTX: Pertussis toxin; PDK-1: 3′-phosphoinositide-dependent kinase-1; PI3-K: Phosphoinositide 3-kinase; PKC: Protein kinase type C; NO-sGC: NO sensitive soluble guanylyl cyclase; TNF-α: tumor necrosis factor-alpha.

## Competing interests

The authors declare that they have no competing interests.

## Authors′ contributions

FPU, CAFA, RFR, RGA, ILB, MJA. FPU, CAFA and RFR participated in the design and performance of tissue cell cultures and proliferation assays. FPU and MJA participated in the design of the study and performed the statistical analysis. FPU and MJA conceived the study. RGA participated in its design and coordination and drafted the manuscript. ILB helped to draft and the final version of the manuscript. All authors read and discussed and approved the final version of this manuscript.

## Authors′ information

FPU: PhD in Cell Biology. Specialist in ASCM and MAPK expert. Actually is a Assistant professor at Biomembranes Section form Instituto de Medicina Experimental (IME). Faculty of Medicine. Central University of Venezuela (UCV).

CAFA: MD and Postgraduate at Biomembranes Section form Instituto de Medicina Experimental (IME). Faculty of Medicine. Central University of Venezuela (UCV).

RFR: MD and Postgraduate at Biomembranes Section form Instituto de Medicina Experimental (IME). Faculty of Medicine. Central University of Venezuela (UCV).

RGA: Ph D. in Biochemistry Molecular and Cell Biology from Cornell University. Actually she is Full Professor in Biochemistry Molecular and Cell Biology at Biomembranes Section form Instituto de Medicina Experimental (IME). Faculty of Medicine. Central University of Venezuela (UCV).

ILB: MD. Specialist in Biochemistry and Cell Biology from UCV. Actually she is Full Professor in Biochemistry and Cell Biology at Biomembranes Section form Instituto de Medicina Experimental (IME). Faculty of Medicine. Central University of Venezuela (UCV).

MJA: MD. & Ph D. in Biochemistry Molecular and Cell Biology from Cornell University. Actually, he is Full Professor in Biochemistry Molecular and Cell Biology at Biomembranes Section and Director of Instituto de Medicina Experimental (IME). Faculty of Medicine. Central University of Venezuela (UCV).
